# Structural injustice, marginality, and neurolaw: a normative comparative and theoretical approach

**DOI:** 10.3389/fsoc.2024.1403914

**Published:** 2024-09-30

**Authors:** José Manuel Díaz-Soto, Diego Borbón

**Affiliations:** ^1^Department of Criminal Law and Criminology, Criminal Law Research Group, Universidad Externado de Colombia, Bogotá, Colombia; ^2^Center for Studies on Genetics and Law, Research Group on Biological Sciences and Law, Universidad Externado de Colombia, Bogotá, Colombia

**Keywords:** neurolaw, neuroethics, prison, mass incarceration, criminal responsibility, poverty, culpability, free will

## Abstract

In this paper, we introduce a perspective based on a comparative viewpoint on the Colombian Penal Code and a theoretical approach to neurolaw and criminal responsibility in contexts of marginality and extreme poverty. We present a further response to the debate on how structural injustice impacts criminal responsibility. By offering a comparative and theoretical insight, this paper enriches the debate and provides an understanding of how legal systems might address these issues. The paper then suggests that other legislations can follow the rule of Article 56 of the Colombian Penal Code, which reduces punishment in circumstances of marginality, ignorance, or extreme poverty. Utilizing neuroscience findings, we briefly highlight the interplay between structural injustice and neurobiological vulnerabilities, emphasizing the complexity of the role of incarceration and criminal law in marginalized populations. We invite scholars to consider debates on alternatives to criminal law, the reduction of prison use and mass incarceration, as well as further remarks on the problem of free will. In this paper, we seek to bridge the gap between neuroscientific insights and socio-legal ethics to foster a more equitable and humane system of justice.

## 1 Introduction

Structural injustice, characterized by discrimination and material inequalities, remains an unresolved issue that deeply influences the criminal justice systems across the world. Its negative effects on marginalized populations are not only socioeconomic but also neuropsychological, leading to a more complex understanding of criminal responsibility. The concept of structural injustice is rooted in the differential impact of social structures, according to Powers (2019), comprising institutions, laws, and informal social practices on individuals based on their membership in various social groups. Powers (2019) identifies the core characteristics of structural injustice as asymmetric, near-inescapable, profound, long-lasting, wide-ranging, and with pervasive impacts that disadvantage certain groups while advantaging others. It impacts wellbeing, increases power imbalances, and reinforces existing inequalities.

In the book “Fair Opportunity and Responsibility,” Brink (2021) argues that punishment and predominant retributivism might seem problematic when considering the offenders who are affected by social injustice and marginality. Retributivism should only punish criminals “proportionate to their desert, that is, to their degree of culpable wrongdoing” (Brink, 2021, p. 149). In essence, marginality might partially impair an agent's capacities that compromise the fair opportunity to avoid wrongdoing by limiting access to the same paths to success as their privileged counterparts. Then, for Brink (2021), the presence of structural injustice has a selective impact on the state's legitimacy to administer punishment as it: “compromises the state's authority to punish crimes by the marginalized that result directly from structural injustice and that this typically provides a partial defense that mitigates, rather than completely exculpates” (p. 211).

In that sense, legal responsibility, according to Brink (2021), implies both normative competence and situational control. First, situational control pertains to an individual's capacity to engage in lawful actions within the context of their circumstances, as they might be affected by necessity or duress. Secondly, normative competence implies a cognitive component that includes the ability to distinguish right from wrong and a volitional component related to forming and executing intentions despite distractions or temptations. For Brink (2021) both are “individually necessary and jointly sufficient for responsibility, because significant impairment of either condition results in an excuse” (p. 158).

## 2 Marginality in Colombia's Penal Code

For Brink (2021) addressing the proper response to offenders who are also victims of social injustice is problematic when highlighting how systemic structural injustice marginalizes individuals with lower socioeconomic status or belonging to discriminated groups, leading to significantly reduced life prospects and opportunities. In that sense, as stated by Brink (2021) “[t]he marginalized in a society do not benefit from the rule of law in the way that other members of their society do, which raises the question of whether we can justify punishing the marginalized in societies that experience structural injustice” (p. 210).

Brink (2021) and Morse (2023) agree that criminal justice might require acknowledging degrees of partial responsibility in punishment, as the current binary approach often leads to disproportionate over-punishment for those with diminished capacities. Then, for Morse (2023) the problem is that with few exceptions “responsibility is bivalent, people are either fully guilty or not, and the responsibility threshold is rather low” (p. 14). In that sense, Morse (2023) argues that “assuming that some form of generic partial responsibility is practically workable, as I do, the questions are how to implement it procedurally and what should be the punishment consequences” (p. 14).

The Colombian Penal Code seems to have a plausible answer that we recommend. Article 56 of Law 599/2000 recognizes that situations of marginality and extreme poverty do not constitute a cause of *justification* in the strict sense, but rather it affects the judgment of reproach: the offender, although responsible, deserves a minor penalty, as a consequence of what could be understood as a diminish culpability. This is interesting because it seems to be in line with what Brink (2021) argues since only exceptional cases are related to behaviors that are unavoidable or impossible to control. Structural injustice does not seem to affect the *wrongful* nature of the action, but rather recognizes that said acts are just significantly *harder* to avoid; not impossible.

In Colombia, the marginality rule of the Penal Code does not intend to recognize that the perpetrator of a crime was unable to act otherwise. On the contrary, the rule outlined in article 56 of the Penal Code acknowledges that the circumstances of marginalization, ignorance, and extreme poverty, associated with the particular crime, significantly influence human behavior, so the punishment might be substantially reduced, but not excluded.

Certainly, as said by Morse (2023) “retributivism requires some conception of partial responsibility and excuse because responsibility is scalar and not bivalent.” In that sense, the rule of marginality of the Colombian Penal Code does not establish a fixed reduction of the sentence or the reproach, but rather, based on the accused crime, it reduces the punitive ranges, so that the judge decides the diminish reproach. As stated in article 56: “will incur a penalty of no more than half of the maximum, nor less than one-sixth of the minimum of that indicated in the respective provision.”

**Figure 1:**
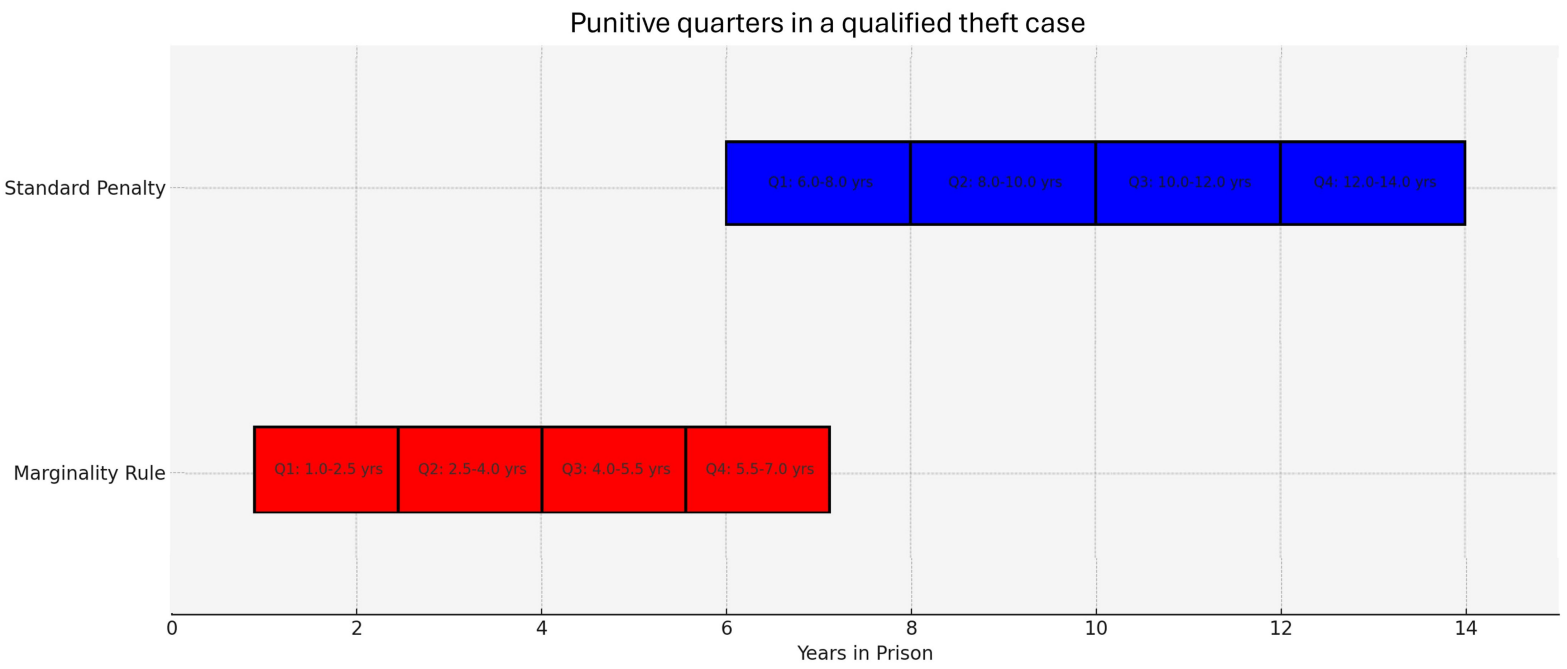
This figure shows the sentencing ranges for a qualified theft case under the Marginality Rule and Standard Penalty in Colombia. The Marginality Rule allows for a sentence between 1 to 7 years, divided into four quarters depending on mitigating and aggravating circumstances. The Standard Penalty ranges from 6 to 14 years, also divided into four quarters.

As a practical example, the current penalty established for qualified theft in Colombia is between 6 and 14 years in prison. With the punitive consequence of the marginality rule, the judge may impose a mitigated sentence between 1 to 7 years. Such consequence allows the judge to assess, for each specific case, the greater or lesser influence of the state of marginality, within the rules that require that the judge must divide the two extremes of the penalty into four quarters, which may be chosen depending on the mitigating and aggravating circumstances accused by the Prosecutor. In Colombia, according to article 61 of the Penal Code Law 599/2000, once the judge has the punitive range of the crime, then must divide these two ranges into four quarters. The first punitive quarter should be chosen if there are no generic aggravating causes; the middle quarters if there are both generic aggravating and mitigating causes; and the last quarter if there are only aggravating factors. Let's now take a look at Figure 1 for the possible punishment for our hypothetical case:

In our hypothetical case of qualified theft with the recognition of the cause of marginality, if the person only acted under generic causes of lesser punishment such as in the absence of criminal records (Art.55.L.1) or acting for noble or altruistic reasons (Art.55.L.2), the penalty would be 1 to 2.5 years (Q.1). Now, under those circumstances together with a generic aggravating factor such as deliberately and inhumanely increasing the suffering of the victim (Art.58.L.8), the penalty would be between 2.5 and 5.5 years (Q.2-Q-3). Finally, if the act is only under aggravated circumstances, the sentence will be between 5.5 and 7 years (Q.4). To choose the penalty within the corresponding punitive quarter, the judge must assess, among other things: the greater or lesser severity of the behavior, the real or potential damage caused, the nature of the factors that aggravate or mitigate the punishment, the intensity of the intention, the presence of recklessness or concurrent negligence, the social need for punishment, and the role it must fulfill in the specific case (Art.61).

In this regard, the Constitutional Court of Colombia. (2019) relates in Judgment SU-479 that ”[w]hoever suffers the circumstances of marginality has a lesser margin of freedom than those of the generality of individuals or a lesser ability to understand the illegality of their behavior.” For its part, the Supreme Court of Justice of Colombia (2019), in SP5356 has determined that marginality “lessens the judgment of reproach that the judge individualizes based on the dogmatic category of culpability, since the circumstances restrict the scope of freedom of the author or participant in a typical and unlawful conduct, in order to motivate himself in accordance with the legal provision so the penalty must also be reduced.” In this sense, both Courts have recognized that extreme marginality affects the capacity to self-determine, but also affects the volitional capacity to motivate oneself according to a cognitive understanding of the law. We advocate for an integral understanding of the impact of marginality, since this circumstance can, to a greater or lesser extent, depending on the case, affect both normative competence and situational control. As Colombian Courts have recognized, this means a form of partial responsibility that affects degrees of freedom required for complete culpability, reducing the judgement of reproach.

In particular, we consider that the Colombian experience can be useful in illuminating possible normative paths in which criminal justice systems can address circumstances of marginality and structural injustice. However, the Colombian experience can also lead to important lessons. As an example, the aforementioned Sentence SU-479/2019 was issued requiring judges to deny the cause of marginality when there is not enough factual support, nor could it be negotiated or agreed with the Prosecutor's Office without support in the case facts, considering that it could discredit justice by significantly reducing the sentence. For this reason, in Colombian legal practice, the application of said cause is rarely recognized, despite the complex social and economic context of Colombia. For example, in 2021 according to the World Bank (2024), Colombia scored a Gini Index of 51.5 which indicates a high level of income inequality, showing a significant wealth disparity between the rich and the poor. In the same way, we consider that in circumstances of marginality, ignorance, and extreme poverty, the role of the State and its justice systems cannot be limited to a punitive response of reduced-time incarceration.

Another unfortunate lesson of Colombia's judicial practice is that the marginality rule as a partial responsibility circumstance, has not been adequately applied by judges in Colombia. For instance, during the period between August 2018 and June 2024, the Criminal Cassation Chamber of the Supreme Court of Justice issued only 27 decisions related to the circumstance of Article 56 CP, and none of these declared a situation constituting marginality or extreme poverty.

However, the Criminal Cassation Chamber has indeed elaborated on the conditions under which the application of Article 56 of the Colombian Penal Code is appropriate. The rule requires, for its configuration, three prerequisites: (1) that the situation of marginality, ignorance, or poverty affecting the perpetrator can be described as “profound” or “extreme,” such that not every socioeconomic disadvantage justifies a reduction in the penalty; (2) that these circumstances have a direct and proximate relation to the commission of the punishable conduct, meaning that they explain the commission or the nature of the criminal offense; and (3) that such circumstances do not constitute a ground for complete exclusion of criminal responsibility, as is the case when the degree of poverty is so severe that it constitutes a ground for absolute exclusion of responsibility due to necessity.

For example, in aforementioned Judgment SP5356-2019, the Chamber reviewed a case involving several economically disadvantaged miners who, lacking the necessary permits, used explosive devices in their mining activities; a conduct constituting the criminal offense of possession of explosives. Applying these criteria to the case under review, the Criminal Cassation Chamber concluded that, although the defendants were under unfavorable economic and social circumstances, these were not severe enough to be considered constitutive of the partial responsibility circumstance of marginality, ignorance, and extreme poverty. Specifically, the Criminal Cassation Chamber considered that the defendants earned a minimum income and had access to some degree of schooling. Instead of recognizing the marginal status of these miners, the Court chose to reduce the penalty due to the lack of knowledge of the illegality of the behavior they were engaging in. As this case, in other 27 opportunities the Supreme Court has analyzed the marginality rule, and in every case, it dismissed its application.

The tendency to not recognize this cause of partial responsibility has been followed by lower-ranking judges. For example, in a ruling on October 6, 2021, the 16th Municipal Criminal Court of Bogotá (2021) convicted a man who had been subjected to permanent and systematic circumstances of marginality and did not grant him the benefits of Article 56. In this process, it was proven that the convicted man had lost his father since he was 3 years old due to violence; that his mother could not raise him at home because she had to work; his brother became a drug addict and his sister a sex worker; he was never able to finish primary school; never had formal training; was also unable to access formal jobs or labor protection; had not contributed to social security, health or a pension; lived in a house of the lowest social stratum and that he was formally registered as living in extreme poverty. Despite this, the aforementioned Court denied the marginality circumstance because it had not been agreed upon in negotiation with the Prosecutor's Office and that recognizing said cause could discredit justice. This is the reality in judicial practice in Colombia as most judges refuse to apply the marginality rule of article 56 of the Penal Code.

## 3 Neurolaw, sociology, and structural injustice

As said by Sifferd (2023), “[t]raditional custodial sentences may just replicate the conditions of structural injustice.” In that sense, it should be considered that by 2021, more than 11 million people were incarcerated in prisons around the world (Fair and Walmsley, 2021), many of whom are marginalized groups. In Colombia, according to the National Penitentiary and Prison Institute (INPEC, 2024), 96.2% of inmates are illiterate, or barely graduated from high school; 0.9% have university undergraduate training, and only 0.3 have postgraduate diplomas. The sociologist Wacquant (2000) is correct in maintaining that prisons are warehouses where society sends the poor and those it considers undesirable.

Furthermore, Mathiesen (2003) warns that no one could seriously affirm that prisons perform resocializing, educational, rehabilitating, reinserting, or repersonalizing functions: “the prison does not have a defense, the prison is a fiasco in terms of its own purposes” (p. 32). Moreover, some judgments rendered by the Constitutional Court of Colombia (1998, 2013, 2015, 2022), including T-153/1998, T-388/2013, T-762/2015, and SU-122/2022, have consistently affirmed the unconstitutional state of affairs in the prison system, and the systematic and extensive infringement of fundamental rights. Also, the Inter-American Court of Human Rights (IACHR, 2022) has ruled condemnations against several nations due to their jails' substandard conditions, including overcrowding and inadequate facilities.

Research in the field of neuropsychology has shown that the neuropsychological wellbeing of individuals is adversely affected by factors such as incarceration, isolation, and degrading prison settings. Research has revealed that only a 3-month period of incarceration is enough to result in significantly diminished self-regulation, heightened propensity for engaging in risky behaviors, and worse cognitive attentiveness (Meijers et al., 2018). This has significance since individuals who have been released from prison may exhibit less capacity to adhere to lawful lifestyles and display an increased inclination toward impulsive behaviors: “In other words, the impoverished environment may contribute to an enhanced risk of reoffending” (Meijers et al., 2018, p. 1).

Prison systems are characterized by a scarcity of physical, mental, and social activity. Now, as said in Borbón (2022), several studies on the negative neuropsychological effects of prison have yielded results that correlate prison with poorer mental health (Haney, 2012; Schnittker et al., 2012; Brinkley-Rubinstein, 2013; Meijers et al., 2015; Constantino et al., 2016). It has also been observed that the prevalence of mental disorders among the incarcerated is considerably greater than in the general population (Durcan and Zwemstra, 2014) and that the number of individuals with mental disorders in prisons in the US is significantly higher than those in mental health institutions (Torrey et al., 2014).

One of the most common mental disorders in prisons is psychopathy and antisocial personality disorder (ASPD). Psychopathy is linked to extensive neuroanatomical and functional brain abnormalities across key regions involved in emotion, cognition, and moral judgment, leading to emotional detachment and empathy deficits (Johanson et al., 2020). According to some studies, psychopathy is the most closely related variable to incarceration, and those who meet the diagnostic criteria are 15 to 25 times more likely to end up incarcerated (Kiehl and Hoffman, 2011). As reviewed by Seid et al. (2022) studies showed the prevalence of ASPD in the prison population is around 35.3% in the USA, 62% in the UK, 56% in Australia, 47% in Nigeria, and 13.6% in Egypt.

Likewise, for Borja and Ostrosky-Solís (2009), there is a complex interaction of genetic, neurobiological, sociocultural, and educational factors in relation to the experience of traumatic events at an early age, with the subsequent development of antisocial personality disorder and psychopathy: the higher the level of victimization, the higher the level of psychopathy in the person. Marginality, violence, and extreme poverty might play an important role in it. And according to Walsh et al. (2013), there is evidence suggesting that “lower socioeconomic-status is generally associated with higher rates of psychopathology, and more specifically with the development and expression of externalizing disorders such as ASPD and psychopathy.”

In this matter, the prevalence of psychopathy can also be addressed by legislation that enshrines a “diminished imputability” model, such as those of some of the penal codes of México^1^, or partially in Spain^2^ which might allow a judgment of lesser reproach, together with a measure of psychological accompaniment (Borbón, 2021a). The current Spanish jurisprudence has understood that personality disorders that should influence criminal responsibility are creditors of an analogical mitigation, resulting in incomplete exemption (diminished imputability), especially when the disorder is of deep severity or is associated with other relevant pathologies, such as chronic or acute alcoholism, intellectual disability, or drug addiction (Supreme Tribunal of Spain, 2005, 2015, 2016), including STS 544/2016, of June 21; 607/2015, of October 9; and 879/2005, of July 4, among others.

In this regard, the issue of criminal policy in contexts of marginality should lead to significant reflections on the role of the State. In this sense, we propose that the discussion should not only be limited to establishing diminished responsibility schemes but should also understand that, in these cases, there must be a significant social and mental health policy. This approach should be aimed at seeking alternatives to imprisonment, establishing substitutes that prioritize alternative, negotiated, and restorative solutions, as well as having significant penitentiary and post-penitentiary programs for education, psychotherapy, and social skills. Mass releases, with reasonable criteria, can be short-term alternatives to ensure dignified living conditions in detention while improving prison environments and modifying the criminal policies of mass incarceration (Díaz Soto and Borbón Rodríguez, 2021).

The scope of neuroscience can and should inform policymaking. Structural injustice, manifesting as poverty, impoverished environments, and lack of opportunities, has been significantly correlated with neurobiological impairments. Research highlights that children living in poverty face detrimental impacts on brain development and mental health, underscoring the critical need for policy interventions. For instance, poverty has been linked to changes in brain structure and function, including reduced volumes in critical areas such as the prefrontal cortex and hippocampus, which are essential for cognitive regulation and emotional control (Lipina and Evers, 2017; Palacios-Barrios and Hanson, 2019). Furthermore, socioeconomic disadvantages contribute to chronic stress, which disrupts neuroendocrine function and impairs cognitive development and executive functioning (Blair and Raver, 2016; Schibli et al., 2017). Longitudinal studies indicate that childhood poverty increases the risk of developing psychopathologies and drug-related issues later in life, revealing the long-term consequences of early deprivation (Manhica et al., 2020; Monk and Hardi, 2023).

In that sense, integrating neuroscientific insights into public policy can inform strategies to mitigate these effects, emphasizing the importance of early interventions and support systems to promote resilience and mental wellbeing in disadvantaged populations (Farah, 2018; Lipina, 2016). But also, the fact that the circumstances of marginality and structural injustice are those that can precisely cause neurobiological dysfunctions correlated with the risk of committing future crimes or engaging in antisocial behavior, are what precisely indicate that the State is in some way co-responsible for generating those risks and this, likewise, reduces the legitimacy of punishing.

## 4 Conclusions for further remarks

The question of criminal responsibility, particularly in contexts of structural injustice, transcends mere legal considerations and delves into realms of neuroscience, morality, and societal structures. In that sense, the field of neurolaw might offer an innovative approach since structural injustice also involves mental health consequences and reflections on the neuropsychological effects of incarceration, thus raising questions on the penal system and the ethical considerations surrounding punishment. In Colombia, the application of the marginality rule within the Penal Code offers a glimpse into the recognition of structural injustice and its impact on antisocial behavior. While it does not exculpate the behavior of the offender, it acknowledges the significant influence of circumstances such as marginalization that affect the margin of freedom of a person.

The insights provided in this paper serve as a catalyst for further debate, toward a legal system that genuinely grapples with the realities of marginalization and the intersection with neurolaw. Although for Morse (2023), talking about free will is a fruitless distraction, we propose to broaden the debates to study alternative approaches to retributive punishment and culpability-based systems. In this sense, the inputs of penal abolitionism in intersection with neurolaw (Borbón, 2022, 2024) or the thesis of hard incompatibilism (Pereboom and Caruso, 2018; Caruso, 2021, 2023) might raise exciting debates on the problem of free will and the neuropsychological effects of prison, which can be useful to abandon paradigms of culpability, retributive justice, and incarceration (Borbón, 2021b, 2022, 2024). As said in Borbón (2022) “under the allegedly false narrative of free will in the penal system, the State ignores the causes of crime by holding the offender responsible and leaving the social structure intact” (p. 3). Thus, the application of neurolaw has the potential to revolutionize, not only the field of criminal justice but the very way in which society understands and approaches the notion of retribution, responsibility, free will, and basic moral desert. Structural injustice, sustained by State neglect, calls for a reconsideration of the legitimacy of criminal justice systems which, rather than contributing to humanity, contribute to human degradation.

## Data Availability

The original contributions presented in the study are included in the article/supplementary material, further inquiries can be directed to the corresponding author.

## Footnotes

^1^In Mexico, reduced imputability is enshrined in most of the Penal Codes of the federal entities, as well as in the Federal Penal Code. The provisions that establish this can be found, for example, in the Codes of the State of Mexico (art. 67), Mexico City (art. 65), Puebla (art. 26), Yucatan (art. 93) and Chihuahua (art. 28), among others.

^2^Article 21 of the Spanish Penal Code recognizes the mitigating and incomplete excuses when the person does not meet all the requirements of grounds of irresponsibility of rules 1, 2 and 3 of article 20 of the Spanish Penal Code.

## References

[B1] Blair C. Raver C. C. (2016). Poverty, stress, and brain development: New directions for prevention and intervention. Acad. Pediatr. 16, S30–S36. 10.1016/j.acap.2016.01.010 27044699 PMC5765853

[B2] Borbón D. (2021a). Trastorno de la personalidad antisocial desde el neuroderecho: responsabilidad penal, libre albedrío y retos de política criminal. Rev. Mexicana Ciencias Penales 4, 187–218. 10.57042/rmcp.v4i13.416

[B3] Borbón D. (2021b). Incompatibilismo humanista: una contrapropuesta del neuroabolicionismo penal. Cuadernos Electr. Filosofía Derecho 45, 46–72. 10.7203/CEFD.45.20713

[B4] Borbón D. (2022). Neurosociology and penal neuroabolitionism: rethinking justice with neuroscience. *Front*. Sociol. 7:814338. 10.3389/fsoc.2022.814338 35146021 PMC8822047

[B5] Borbón D. (2024). Free will, quarantines, and moral enhancements: neuroabolitionism as an alternative to criminal law. *Front*. Sociol. 9:1395986. 10.3389/fsoc.2024.1395986 38855009 PMC11157510

[B6] Borja K. Ostrosky-Solís F. (2009). Los eventos traumáticos tempranos y su relación con la psicopatía criminal [Early traumatic events and its relationship with criminal psychopathy]. Rev. Chil. Neuropsico. 4, 160–169.

[B7] Brink D. (2021). Fair Opportunity and Responsibility. Oxford: Oxford University Press. 10.1093/oso/9780198859468.001.0001

[B8] Brinkley-Rubinstein L. (2013). Incarceration as a catalyst for worsening health. Health Justice 1:3. 10.1186/2194-7899-1-334845673

[B9] Caruso G. D. (2021). Rejecting Retributivism: Free Will, Punishment, and Criminal Justice. Cambridge: Cambridge University Press. 10.1017/9781108689304

[B10] Caruso G. D. (2023). “Retributivism, free will, and the public health-quarantine model,” in The Palgrave Handbook on the Philosophy of Punishment. Palgrave Handbooks in the Philosophy of Law, ed. M.C. Altman (Cham: Palgrave Macmillan). 10.1007/978-3-031-11874-6_22

[B11] Constantino P. Assis S. G. Pinto L. W. (2016). The impact of prisons on the mental health of prisoners in the state of Rio de Janeiro, Brazil. Cien. Saude Colet. 21, 2089–2100. 10.1590/1413-81232015217.01222016 27383343

[B12] Constitutional Court of Colombia (1998). Judgment T-153 of 1998.

[B13] Constitutional Court of Colombia (2013). Judgment T-388 of 2013.

[B14] Constitutional Court of Colombia (2015). Judgment T-762 of 2015.

[B15] Constitutional Court of Colombia (2019). Judgment SU-479 of 2019.

[B16] Constitutional Court of Colombia (2022). Judgment SU-122 of 2022.

[B17] Díaz Soto J. M. Borbón Rodríguez D. A. (2021). “La imperiosa necesidad de reducir la población penitenciaria en Colombia: Análisis crítico de la declaratoria del estado de cosas inconstitucional en las cárceles y penitenciarias del país,” in Criminalización y control: retos hacia visiones restaurativas e interculturales de la justicia, eds. M. Gutiérrez, and M. Olarte (Bogotá: Universidad Externado de Colombia), 47–84.

[B18] Durcan G. Zwemstra J. (2014). “Mental health in prison,” in Prisons and Health, eds. S. Enggist, L. Møller., G. Galea., and C. Udesen (Copenhagen: World Health Organization).

[B19] Fair H. Walmsley R. (2021). World Prison Population List. Thirteenth Edition. London: Institute for Crime and Justice Policy Research at Birkbeck University of London. Available at: https://www.prisonstudies.org/sites/default/files/resources/downloads/world_prison_population_list_13th_edition.pdf (accessed March 18, 2024).

[B20] Farah M. J. (2018). Socioeconomic status and the brain: prospects for neuroscience-informed policy. Nat. Rev. Neurosci. 19, 428–438. 10.1038/s41583-018-0023-2 29867123

[B21] Haney C. (2012). “The psychological effects of imprisonment,” in The Oxford Handbook of Sentencing and Corrections (Oxford: Oxford University Press). 10.1093/oxfordhb/9780199730148.013.0024

[B22] IACHR (2022). Cuadernillo No. 9 de Jurisprudencia de la Corte Interamericana de Derechos Humanos, Personas privadas de libertad.

[B23] INPEC (2024). Statistical Boards - Intramural academic level. Available at: https://www.inpec.gov.co (accessed March 20, 2024).

[B24] Johanson M. Vaurio O. Tiihonen J. Lähteenvuo M. (2020). A systematic literature review of neuroimaging of psychopathic traits. *Front*. Psychiatry 10:1027. 10.3389/fpsyt.2019.01027 32116828 PMC7016047

[B25] Kiehl K. A. Hoffman M. B. (2011). The criminal psychopath: history, neuroscience, treatment, and economics. Jurimetrics 51, 355–397. 24944437 PMC4059069

[B26] Lipina S. J. (2016). The biological side of social determinants: neural costs of childhood poverty. PROSPECTS 46, 265–280. 10.1007/s11125-017-9390-0

[B27] Lipina S. J. Evers K. (2017). Neuroscience of childhood poverty: Evidence of impacts and mechanisms as vehicles of dialog with ethics. Front. Psychol. 8:61. 10.3389/fpsyg.2017.00061 28184204 PMC5266697

[B28] Manhica H. Straatmann V. S. Lundin A. Agardh E. Danielsson A.-K. (2020). Association between poverty exposure during childhood and adolescence, and drug use disorders and drug-related crimes later in life. Addiction 116, 569–578. 10.2139/ssrn.3559619 33197093 PMC8247994

[B29] Mathiesen T. (2003). Juicio a la prisión: una evaluación crí*tica*. Buenos Aires: EDIAR.

[B30] Meijers J. Harte J. M. Jonker F. A. Meynen G. (2015). Prison brain? Executive dysfunction in prisoners. *Front*. Psychol. 6:43. 10.3389/fpsyg.2015.00043 25688221 PMC4311616

[B31] Meijers J. Harte J. M. Meynen G. Cuijpers P. Scherder E. J. A. (2018). Reduced self-control after 3 months of imprisonment; a pilot study. *Front*. Psychol. 9:69. 10.3389/fpsyg.2018.00069 29449824 PMC5799890

[B32] Monk C. S. Hardi F. A. (2023). Poverty, brain development, and mental health: progress, challenges, and paths forward. Ann. Rev. Dev. Psychol. 5, 309–330. 10.1146/annurev-devpsych-011922-012402

[B33] Morse S. J. (2023). “Criminal responsibility reconsidered,” in Criminal Law, Philosophy. 10.1007/s11572-023-09702-7

[B34] Municipal Criminal Court of Bogotá (2021). Ruling of October 6, 2021. C.U.I 110016000019202100410

[B35] Palacios-Barrios E. E. Hanson J. L. (2019). Poverty and self-regulation: connecting psychosocial processes, neurobiology, and the risk for psychopathology. Compr. Psychiatry 90, 52–64. 10.1016/j.comppsych.2018.12.012 30711814

[B36] Pereboom D. Caruso G. D. (2018). “Hard-incompatibilist existentialism: Neuroscience, punishment, and meaning in life,” in Neuroexistentialism: Meaning, morals, and purpose in the age of neuroscience, eds. G. D. Caruso and O. Flanagan (Oxford: Oxford University Press), 193–222.

[B37] Powers M. (2019). What structural injustice is. In *Structural Injustice: Power, Advantage, and Human Rights*. New York: Oxford University Press. 10.1093/oso/9780190053987.001.0001

[B38] Schibli K. Wong K. Hedayati N. D'Angiulli A. (2017). Attending, learning, and socioeconomic disadvantage: developmental cognitive and social neuroscience of resilience and vulnerability. Ann. N. Y. Acad. Sci. 1396, 19–38. 10.1111/nyas.13369 28548461

[B39] Schnittker J. Massoglia M. Uggen C. (2012). Out and down: incarceration and psychiatric disorders. J. Health Soc. Behav. 53, 448–464. 10.1177/0022146512453928 23197484

[B40] Seid M. Anbesaw T. Melke S. Beteshe D. Mussa H. Asmamaw A.. (2022). Antisocial personality disorder and associated factors among incarcerated in prison in Dessie city correctional center, Dessie, Ethiopia: a cross-sectional study. BMC Psychiat. 22:53. 10.1186/s12888-022-03710-y 35073903 PMC8785502

[B41] Sifferd K. (2023). “How does structural injustice impact criminal responsibility?” in Criminal Law, Philosophy. 10.1007/s11572-023-09697-1

[B42] Supreme Court of Justice of Colombia Criminal Cassation Chamber. (2019). Judgment SP5356-2019 – 50525 of the 4^th^ of December 2019.

[B43] Supreme Tribunal of Spain (2005). Ruling No. 879/2005 of July 4.

[B44] Supreme Tribunal of Spain (2015). Ruling No. 607/2015 of October 9.

[B45] Supreme Tribunal of Spain (2016). Ruling No. 544/2016 of June 21.

[B46] Torrey E. F. Zdanowicz M. T. Kennard A. D. Lamb H. R. Eslinger D. F. Biasotti M. C.. (2014). The Treatment of Persons with Mental Illness in Prisons and Jails: A State Survey. Arlington, VA: Treatment Advocacy Center.

[B47] Wacquant L. (2000). Las cárceles de la miseria. Buenos Aires: Editorial Manantial.

[B48] Walsh Z. Shea M. T. Yen S. Ansell E. B. Grilo C. M. McGlashan T. H.. (2013). Socioeconomic-status and mental health in a personality disorder sample: the importance of neighborhood factors. J. Pers. Disord. 27, 820–831. 10.1521/pedi_2012_26_061 22984860 PMC4628287

[B49] World Bank (2024). Gini index - Colombia. The World Bank Group. Available at: https://data.worldbank.org/indicator/SI.POV.GINI?locations=CO (accessed March 18, 2024).

